# The Human Frontal Oculomotor Cortical Areas Contribute Asymmetrically to Motor Planning in a Gap Saccade Task

**DOI:** 10.1371/journal.pone.0007278

**Published:** 2009-09-30

**Authors:** Paul van Donkelaar, Yu Lin, David Hewlett

**Affiliations:** Department of Human Physiology and Institute of Neuroscience, University of Oregon, Eugene, Oregon, United States of America; University of Regensburg, Germany

## Abstract

**Background:**

Saccadic eye movements are used to rapidly align the fovea with the image of objects of interest in peripheral vision. We have recently shown that in children there is a high preponderance of quick latency but poorly planned saccades that consistently fall short of the target goal. The characteristics of these multiple saccades are consistent with a lack of proper inhibitory control of cortical oculomotor areas on the brainstem saccade generation circuitry.

**Methodology/Principal Findings:**

In the present paper, we directly tested this assumption by using single pulse transcranial magnetic stimulation (TMS) to transiently disrupt neuronal activity in the frontal eye fields (FEF) and supplementary eye fields (SEF) in adults performing a gap saccade task. The results showed that the incidence of multiple saccades was increased for ispiversive but not contraversive directions for the right and left FEF, the left SEF, but not for the right SEF. Moreover, this disruption was most substantial during the ∼50 ms period around the appearance of the peripheral target. A control condition in which the dorsal motor cortex was stimulated demonstrated that this was not due to any non-specific effects of the TMS influencing the spatial distribution of attention.

**Conclusions/Significance:**

Taken together, the results are consistent with a direction-dependent role of the FEF and left SEF in delaying the release of saccadic eye movements until they have been fully planned.

## Introduction

Saccades occur as the result of an interaction between high-level decision-making areas in the cortex and lower level subcortical saccade execution circuits [1]–[4]. The frontal and supplementary eye fields (FEF/SEF), in particular, have been shown to contribute to controlling saccade-related activity in the superior colliculus (SC) and, thus, the occurrence of the saccade [5]. Moreover, this control is the result of an appropriate balance of activation across these frontal oculomotor areas within each hemisphere [6]. The goal of these planning and decision-making processes is to produce a saccade with the quickest reaction time and the most accuracy possible so that the object of interest can be foveated appropriately. This is especially relevant in the real world where multiple objects may compete for selection [7].

A typical saccade consists of a single primary change in eye position that covers all or most of the distance to the target, followed shortly thereafter by a small secondary corrective eye movement if required. A much less common form of output consists of a series of at least two smaller saccades occurring successively termed a multiple saccade [8]. Because multiple saccades occur so infrequently in healthy young adults, they are typically considered an error and discarded from further analysis. An alternative explanation, however, is that this type of output reflects a more automatic form of saccade execution that occurs before planning is fully complete. In a recent developmental study, we demonstrated that although the frequency of this type of saccade decreases substantially from 4 years of age to adulthood, the rapidity with which it is generated remains remarkably invariant across age groups [9]. This suggests that multiple saccades represent a form of oculomotor planning that occurs without coordinated input from the cerebral cortex; reflecting instead a more automatic form of output by the midbrain and brainstem saccade generation circuitry – structures that are well developed within the first decade of life.

In the current study, we used transcranial magnetic stimulation (TMS) to examine this issue more directly. TMS can be used to probe whether a specific brain region is necessary for normal task performance. It is thought to disrupt the pattern of activity normally associated with task performance and, thus, result in subtle but systematic alterations in behavior. In the current study, we used single pulse TMS to transiently disrupt neuronal activity in the FEF or SEF of healthy young adults while they performed a gap saccade task. We predicted that if the FEF or SEF were directly involved in controlling the release of saccades, then disrupting these areas with TMS should lead to an increased preponderance of multiple saccades.

## Materials and Methods

### Participants

Nineteen subjects participated – 9 males and 10 females (mean age 24.7 years). All had normal or corrected-to normal vision including binocular stereoscopic vision and no sensory, motor, cognitive, or attentional deficits that would affect saccadic eye movements.

### Ethics Statement

All subjects signed an informed consent form prior to participation and the University of Oregon Office for Protection of Human Subjects approved the experimental protocol.

### Transcranial Magnetic Stimulation

**Figure 1:**
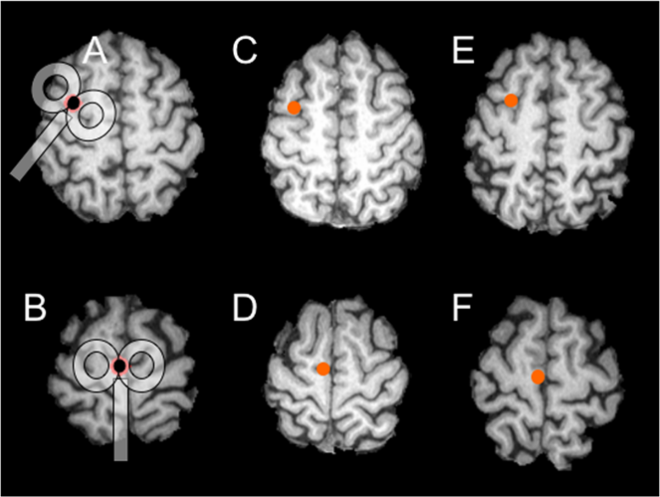
Reconstructed TMS sites from 3 participants. Stimulation sites over the FEF (A, C, E) and SEF (B, D, F) were reconstructed by localizing the position of a high-intensity signal marker with respect to the underlying sulcal anatomy. The TMS coil is drawn in (A,B) to indicate orientation at each stimulation site.

A 2T Magstim 200 was used to deliver single pulses of TMS through a figure eight coil. TMS stimulation intensity was determined by first determining the motor threshold for each subject after localizing the hot point of the hand region in the motor cortex. The motor threshold was defined as the lowest current intensity at which an observable twitch of the first dorsal interosseus (FDI) of the contralateral hand could most reliably be evoked by TMS. During the experimental sessions, the stimulator output was set to 110% of the motor threshold and delivered over either the right or left FEF; right or left SEF, or left dorsal motor cortex. Based on previous reports the FEF was localized ∼2 cm anterior to the motor hot point [10]–[12] and the SEF ∼3 cm anterior to the vertex and 0.5 cm lateral to the midline [13]. Finally, the left dorsal motor area was used as a control site and located 1 cm lateral to the vertex. For the FEF and dorsal motor sites, the stimulating coil was oriented at a 45-degree angle to the midline with the handle pointing in the posterior direction, whereas for the SEF site, the stimulating coil was oriented parallel to the midline with the handle pointing towards the back of the head (see Figure 1). Participants wore a swim cap on which markings were made to facilitate stimulator localization. The coil was held in place with a clamping system and the head was stabilized with a chin rest. None of the subjects reported any undesirable side effects resulting from the stimulation. Confirmation of the experimental stimulation sites was carried out in three of the participants. For this purpose, structural MRIs were recorded with high-contrast markers placed at each of the stimulation sites. During the scanning, the head of the subject was positioned comfortably within the head coil, and head motion was minimized with foam padding. In addition, participants wore earplugs and headphones to protect their hearing. Whole-brain anatomical scans were collected using a T1-weighted magnetization-prepared rapid gradient-echo sequence (time repetition  = 2500 ms, echo time  = 4.38 ms, flip angle  = 8°, field of view  = 256×256 mm; 176 slices per slab at 1 mm slice thickness). Figure 1 shows the reconstructed stimulation sites over the left FEF and SEF from each of the participants. In each case, the FEF marker aligned with the junction of the superior frontal and precentral sulcus (Fig. 1A, C, E); whereas the SEF marker was localized just off the midline adjacent to the upper region of the paracentral sulcus (Fig. 1B, D, F). The average MNI coordinates across the three participants for each stimulation site were as follows: right FEF (x = 33, y = −2, z = 52); left FEF (x = −34, y = −3, z = 53); right SEF (x = 2, y = 0, z = 62); left SEF (x = −3, y = 1, z = 60). These are in close proximity to the sites used in several recent TMS studies in which the FEF [14]–[16] and SEF [13], [17], [18] were targeted.

### Experimental Task

The experimental task consisted of the subject making a series of horizontal saccades to a target presented on a display screen. At the start of each trial, a target (a “plus” sign subtending ∼0.5°) appeared at the center of the display screen. This target then disappeared and 200 ms later a peripheral target (a circle subtending ∼0.5°) appeared 5° or 10° to the left or right of the original central target. The target remained at the new location for 500 ms before jumping back to the center position. The goal for the participant was to follow the target jumps with their eyes by making saccades and maintaining fixation on the target. On 83% of the trials a single pulse of TMS was delivered. When TMS was delivered, the subsequent trial was initiated after a delay of 7 seconds. Participants completed five blocks of 48 trials. Each block consisted of six different TMS trial types: no TMS, TMS applied 50 or 100 ms before and after the peripheral target onset, and TMS applied at target onset. These were combined with the 4 different target directions/amplitudes resulting in a total of 10 trials for each possible trial combination. Fourteen subjects completed four separate counterbalanced sessions separated by at least 7 days in which either the left or right FEF or SEF was stimulated during the task. Five additional subjects completed a separate condition in which the left dorsal motor cortex was stimulated as a control site.

### Data Recording and Analysis

Horizontal eye movements were monitored using an infrared corneal reflection device (Skalar IRIS). The device was calibrated prior to each block of trials by having the subject fixate on a series of targets at known eccentricities. This system provided a signal proportional to the position of the eye with respect to the head with an optimal resolution of 2 min arc and linearity within 3% between −25 deg and +25 deg. The eye movement recordings were analyzed using a graphical user interface implemented in Matlab. Saccadic reaction time (SRT) and frequency of multiple saccades were the two dependent variables of interest. SRT was defined as the period of time from the appearance of the peripheral target to the onset of a detectable change in eye position. The onset was determined by the program when eye velocity was greater than 30°/s for a duration greater than 10 ms. The user could subsequently adjust the automatically determined onset if it was judged to be inaccurate. Multiple saccades were defined as two or more changes in eye position in which the initial saccade covered <90% of the distance to the target [9]. Single saccades were defined as one discrete change in eye position covering ≥90% of the distance to the target. We calculated the frequency of multiple saccade occurrence during trials with TMS relative to the trials without TMS. Trials with large artifacts resulting from blinking, head movement, or equipment malfunction that prevented eye measurement were removed from analysis. Together, these anomalous trials accounted for less than 5% of the data overall. Repeated measures analyses of variance were used to assess the statistical significance of the different effects for each dependent variable. For SRT, we used a 2 (saccade type: single vs. multiple) ×2 (saccade direction: ipsiversive vs. contraversive to TMS site) ×5 (TMS delay: −100, −50, 0, 50, 100 ms) ANOVA for each stimulation site. For the frequency of multiple saccades, we used a 2 (saccade direction: ipsiversive vs. contraversive to TMS site) ×5 (TMS delay: −100, −50, 0, 50, 100 ms) ANOVA for each stimulation site. Post-hoc Tukey's tests were used to examine the locus of any main effects or interactions.

## Results

**Figure 2:**
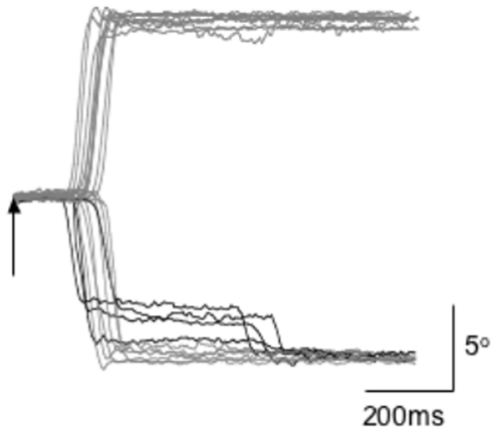
Saccadic eye movements are influenced by TMS delivered over the ipsilateral frontal oculomotor cortical areas. Saccade traces for a single subject aligned to the appearance of the peripheral target and delivery of TMS to the left FEF (vertical arrow). TMS led to the generation of multiple saccades (black traces) when the peripheral target appeared ipsiversive, but not contraversive, to the site of stimulation (leftward is downward in these traces).

**Figure 3:**
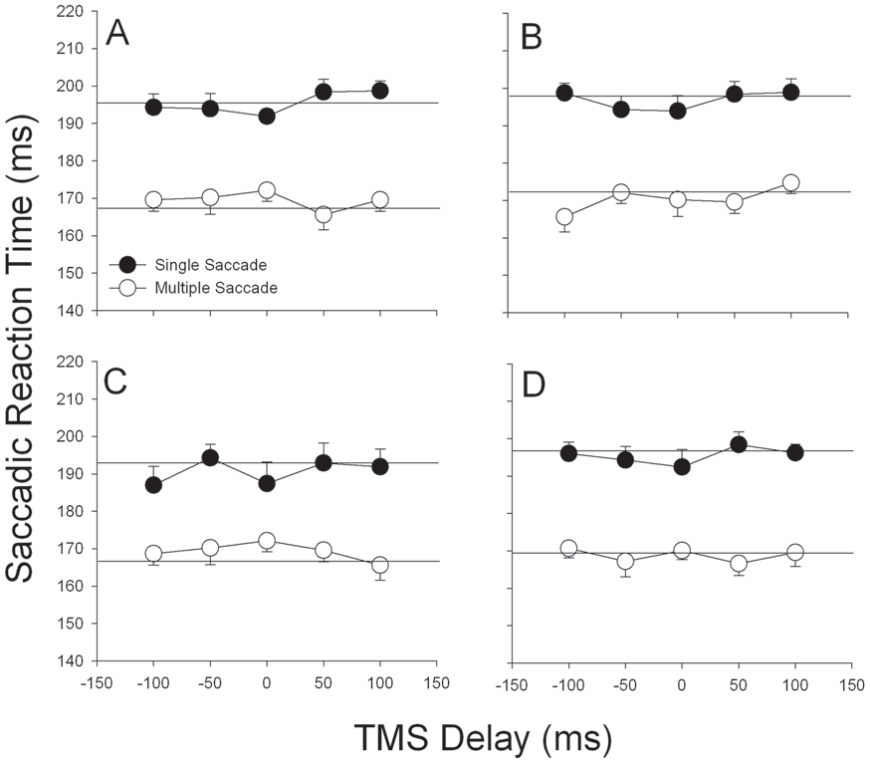
Reaction time is faster during multiple compared to single saccades. Group means for saccade reaction time for single (*black*) and multiple (*white*) saccades when TMS was delivered over the left FEF (A), right FEF (B), left SEF (C), and right SEF (D). Horizontal lines represent mean SRT during the interleaved trials without TMS. These vary across the 4 conditions because each was performed in a separate session. *Error bars*, 1 intersubject SE.

Figure 2 shows a series of saccade traces produced by a single subject during trials with TMS delivered to the left FEF coincident with the appearance of the peripheral target. During the trials without TMS (not shown), this subject always made a single saccade which covered most or all of the distance to the peripheral target. However, when TMS was delivered coincident with peripheral target appearance, there was a tendency to produce multiple saccades to the ipsiversive, but not contraversive, side relative to the site of stimulation. The multiple saccades were characterized by shorter latencies and a relatively small amplitude initial saccade followed by a second saccade to foveate the target [9]. The reduction in SRT for multiple saccades is shown for each of the experimental TMS sites in Figure 3 collapsed across saccade direction. Analyses of variance revealed a significant saccade type effect at each site (Left FEF: F[1,279] = 150.76, p<0.001; Right FEF: F[1,279] = 98.35, p<0.001; Left SEF: F[1,279] = 104.35, p<0.001; Right SEF: F[1,279] = 135.23, p<0.001) but no effect of TMS delay, saccade direction, or any interactions between these three variables. A similar pattern of results was observed in the dorsal motor cortex control site (Saccade type effect: F[1,99] = 89.48, p<0.001).

**Figure 4:**
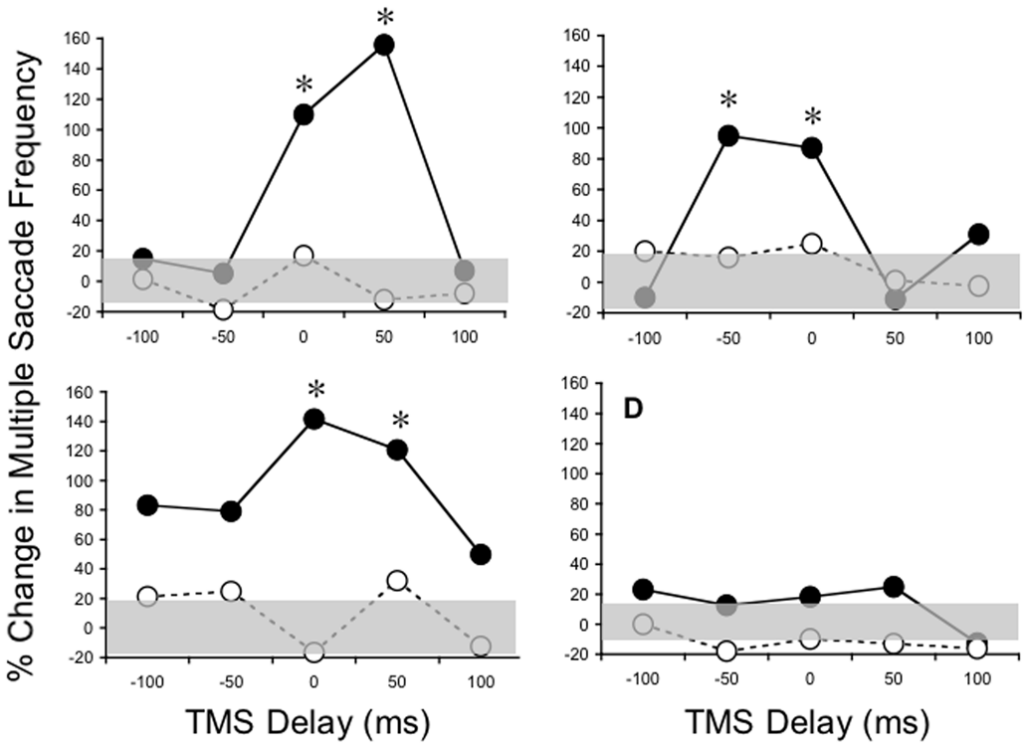
TMS leads to significant increases in multiple saccade frequency ipsilateral to the stimulation site. Percentage change in multiple saccade frequency plotted as a function of TMS delivery time for the left FEF (A), right FEF (B), left SEF (C), and right SEF (D). *Solid symbols* represent saccades made ipsiversive to the side of stimulation; *open symbols*, contraversive saccades. Shaded region centered on 0 represents the intersubject variability in multiple saccade frequency during the non-TMS trials. This varies across the 4 conditions because each was performed in a separate session. Asterisks, significant difference between ipsiversive and contraversive multiple saccade frequency.

The tendency for more multiple saccades to be generated ipsiversive to the site of stimulation is captured in Figure 4 which displays the percentage change in the frequency of multiple saccades as a function of the time at which TMS is delivered. For the FEF (Fig. 4A,B) there is a clear increase in multiple saccade frequency for saccades directed ipsiversively, but not contraversively when TMS is delivered to either the left (main effect of saccade direction: F[1,139] = 4.19, p = 0.018) or right (main effect of saccade direction: F[1,139] = 2.41, p = 0.0342) hemisphere. Post-hoc tests demonstrated that this effect was driven by differences across ipsi- versus contraversive saccades when TMS was delivered coincident with peripheral target appearance or 50 ms later in the left FEF (p<0.05), or coincident with peripheral target appearance or 50 ms earlier in the right FEF (p<0.05). This trend was partially replicated in the SEF (Fig. 4C,D). In particular, there was a significant effect of saccade direction when TMS was delivered to the left SEF (F[1,139] = 2.95, p = 0.0396), but not when it was delivered to the right SEF (F[1,139] = 0.55, p = 0.261). Again, post-hoc tests revealed that this was due to differences between ipsi- versus contraversive saccades when TMS was delivered over the left SEF coincident with the appearance of the peripheral target or 50 ms later (p<0.05). Finally, stimulation at the dorsal motor cortex control site did not lead to any change in the incidence of multiple saccades across ipsiversive and contraversive directions (F[1,49] = 0.55, p = 0.261). Thus, taken together, these data demonstrate that whereas the reaction times of multiple saccades remained invariant across the different combinations of trial types; the frequency with which they occurred was systematically influenced by both TMS delay and saccade direction.

## Discussion

Saccadic eye movements are generated many times each day to foveate objects of interest in the field of view. The basic characteristics of saccades in adults are well known – they have very high velocities that are linearly related to amplitude for saccades up to 50° [19]. Saccade execution occurs as the result of a pulse and step in the activation of brainstem ocular motor neurons. The pulse drives the eye to the new position and the step holds it at this location [20]. This systematic modulation of the brainstem saccade generation circuits is controlled by the balance of activation in fixation and saccade cells in the superior colliculus (SC). When fixation cell activity is high, saccade cell activity is low and the eyes remain stable. However, when saccade cell activity increases, there is a concomitant decrease in activity in the fixation cells and, as a result, a saccade is generated [21]. This release from fixation based on the balance of activation in fixation and saccade cells in the SC is the basis by which saccade control occurs. Interestingly, however, if the only neural processing occurring prior to saccade execution was that associated with initially identifying the target visually and then generating the appropriate motor output, saccadic reaction time (SRT) would be around 60 ms. Yet, studies have shown that SRT is typically around 200 ms in duration. This difference in time is thought to reflect additional processing associated with planning and decision-making occurring most prominently in the FEF and SEF [7]. The FEF in particular sends direct excitatory and inhibitory signals to the SC that control the balance of activity in the fixation and saccade cells and, thus, can in theory provide full control of whether or not a saccade can be made [5]. The goal of these planning and decision-making processes is to produce a saccade with the quickest SRT and the most accuracy possible so that the object of interest can be foveated appropriately.

Multiple saccades are a form of saccadic output characterized by short latencies and hypometric amplitudes falling less than 90% of the distance to the target. We have shown previously that multiple saccades occur much more frequently in young adolescents than in older children or adults [9]. However, the latencies of multiple saccades remain remarkably invariant across development. Based on this and other related evidence, we have suggested that multiple saccades reflect a release from fixation within the midbrain and brainstem prior to the completion of planning and decision-making at the level of the cortex. Indeed, it has been suggested that the purpose of the descending input from the cortex is to procrastinate so that accurately planned responses are elicited [22], [23] - the implication being that saccades that are released too soon are more likely to be inaccurate in some way.

This hypothesis was tested directly in the present experiment by using TMS to transiently disrupt processing in either the FEF or SEF during saccade preparation in adults performing a gap paradigm. We found that multiple saccades were more likely to occur for targets directed ipsiversive, but not contraversive, to the stimulation site for both the right and left FEF and left SEF. This implies that the FEF, and to a lesser extent the SEF, contributes directly to the procrastination required to generate accurate saccades. When the processing occurring at these sites was disrupted with TMS, multiple saccades were much more likely to occur, suggesting that the appropriate balance of activation in the left and right frontal oculomotor areas is required to properly inhibit the brainstem saccade generation circuitry until the saccade is fully planned. If this balance is perturbed, the implication is that the brainstem is released from the descending cortical control too early. These findings are consistent with results showing that patients with FEF lesions are more likely than controls to make quick latency express saccades in a gap paradigm [24].

The direction-dependence of the TMS effect suggests that the FEF and SEF contribute more substantially to the procrastination required during ipsiversive than contraversive saccades. This finding is inconsistent with microstimulation [25] and single-unit recording [26] studies in nonhuman primates and brain imaging studies in humans [27] which demonstrate mainly contraversive saccade-related activity in the FEF and SEF. Schlag and colleagues [6] demonstrated that cells in the FEF are modulated by microstimulation in the homonymous region of the FEF in the opposite hemisphere and that this can alter the characteristics of subsequently generated saccades. The fact that TMS had no effect on contraversive saccades, but increased inappropriately-generated multiple saccades ipsiversive to the stimulation site implies that this form of stimulation very likely biases the balance between left and right hemispheres by disrupting the pattern of activation preferentially in the targeted hemisphere and, thus, allowing that in the opposite hemisphere to dominate any descending input. Based on this reasoning, it follows that the greater contribution from the contralateral hemisphere when TMS is delivered leads to an increased preponderance of multiple saccades. This implies that the normal balance between ipsilateral and contralateral frontal oculomotor cortical sites is required to properly inhibit the release from fixation until the saccade parameters are appropriately specified.

We observed this effect in the left and right FEF as well as the left SEF. The fact that it was not observed with right SEF stimulation implies either that the right SEF does not play a role in this aspect of saccade preparation or that our stimulation was not accurately directed at the right SEF. To our knowledge, most available evidence examining the contribution of the SEF to saccade control has not demonstrated a functional asymmetry between left and right hemispheres. Thus, the most plausible and conservative explanation for the current results appears to be a mislocalization in our attempts to target the right SEF. However, at the same time, it seems unlikely that any mislocalization would be consistently limited to the right SEF as opposed to distributed across each of the other targeted sites. Therefore, the current results may also be interpreted as representing a real functional difference related to saccade control between the left and right SEF. Future studies should be carried out to more explicitly test this notion.

In addition to the directional effect, there was also ∼50 ms period around the appearance of the peripheral target during which stimulation was most effective. Later or earlier stimulation times did not lead to a similar increase in the incidence of ipsiversive multiple saccades. This finding suggests that there is a critical time window during which the balanced activation across the hemispheres is vitally important. This timing was relatively constant across the three areas which demonstrated the effect and is consistent with nonhuman primate studies [25], [26]. The fact that this timing was temporally proximate to the appearance of the peripheral target rather than to the saccadic output itself implies that we mainly affected the pattern of activation associated with visuomotor as opposed to purely motor processing – an implication which is supported by previous TMS studies which more directly examined the contribution of frontal oculomotor areas to visual attention and target discrimination [28], [29].

In conclusion, we have directly demonstrated using TMS that the multiple saccades we have previously characterized in young children [9] can be induced in adults by perturbing the balance in activity between frontal cortical oculomotor sites; most likely resulting in inappropriate inhibitory control of the brainstem saccade generation circuitry. Furthermore, we have shown that these inhibitory processes are direction- and time-dependent being maximally perturbed by the TMS during the ∼50 ms period surrounding the appearance of the saccade target ipsiversive to the site of stimulation. Taken together, these findings provide direct confirmation that the human FEF and SEF are intimately involved in the procrastination required to generate accurate saccades.

## References

[1] Bisley JW, Goldberg ME (2003). The role of the parietal cortex in the neural processing of saccadic eye movements.. Adv Neurol.

[2] Isa T, Kobayashi Y (2004). Switching between cortical and subcortical sensorimotor pathways.. Prog Brain Res.

[3] Pierrot-Deseilligny C, Milea D, Muri, RM (2004). Eye movement control by the cerebral cortex.. Curr Opin Neurol.

[4] Neggers SF, Raemakers MA, Lampmann EE, Postma A, Ramsey NF (2005). Cortical and subcortical contributions to saccade latency in the human brain.. Eur J Neurosci.

[5] Munoz DP, Everling S (2004). Look away: the anti-saccade task and the voluntary control of eye movement.. Nat Rev Neurosci.

[6] Schlag J, Dassonville P, Schlag-Rey M (1998). Interaction of the two frontal eye fields before saccade onset.. J Neurophysiol.

[7] Schall JD (2004). On building a bridge between brain and behavior.. Annu Rev Psychol.

[8] Salapatek P, Aslin RN, Simonson J, Pulos E (1980). Infant saccadic eye movements to visible and previously visible targets.. Child Dev.

[9] van Donkelaar P, Saavedra S, Woollacott M (2007). Multiple saccades are more automatic that single saccades.. J Neurophysiol.

[10] Nyffeler T, Wurtz P, Lüscher HR, Hess CW, Senn W (2006). Repetitive TMS over the human oculomotor cortex: comparison of 1-Hz and theta burst stimulation.. Neurosci Lett.

[11] Müri RM, Hess CW, Meienberg O (1991). Transcranial stimulation of the human frontal eye field by magnetic pulses.. Exp Brain Res.

[12] Ro T, Cheifet S, Ingle H, Shoup R, Rafal R (1999). Localization of the human frontal eye fields and motor hand area with transcranial magnetic stimulation and magnetic resonance imaging.. Neuropsychologia.

[13] Nyffeler T, Rivaud-Pechoux S, Wattiez N, Gaymard B (2008). Involvement of the supplementary eye field in oculomotor predictive behavior.. J Cogn Neurosci.

[14] Campana G, Cowey A, Casco C, Oudsen I, Walsh V (2007). Left frontal eye field remembers “where” but not “what.. Neuropsychologia.

[15] Juan CH, Muggleton NG, Tzeng OJL, Hung DL, Cowey A (2008). Segregation of visual selection and saccades in human frontal eye fields.. Cereb Cortex.

[16] Prime SL, Vesia M, Crawford JD (2009). TMS over human frontal eye fields disrupts trans-saccadic memory of multiple objects.. Cereb Cortex.

[17] Drew AS, van Donkelaar P (2007). The contribution of the human FEF and SEF to smooth pursuit initiation.. Cereb Cortex.

[18] Gagnon D, Paus T, Grosbras MH, Pike GB, O'Driscoll GA (2006). Transcranial magnetic stimulation of frontal oculomotor regions during smooth pursuit.. J Neurosci.

[19] Girard B, Berthoz A (2005). From brainstem to cortex: computational models of saccade generation circuitry.. Prog Neurobiol.

[20] Scudder CA, Kaneko CS, Fuchs AF (2002). The brainstem burst generator for saccadic eye movements: a modern synthesis.. Exp Brain Res.

[21] Enderle JD (2002). Neural control of saccades.. Prog Brain Res.

[22] Carpenter RHS (2004). Contrast, probability, and saccadic latency; evidence for independence of detection and decision.. Curr Biol.

[23] Reddi BA, Carpenter RH (2000). The influence of urgency on decision time.. Nat Neurosci.

[24] Braun D, Weber H, Mergner T, Schulte-Monting J (1992). Saccadic reaction times in patients with frontal and parietal lesions.. Brain.

[25] Izawa Y, Suzuki H, Shinoda Y (2004). Suppression of visually and memory-guided saccades induced by electrical stimulation of the monkey frontal eye field. I. Suppression of ipsilateral saccades.. J Neurophysiol.

[26] Everling S, Munoz DP (2000). Neuronal correlates for preparatory set associated with pro-saccades and anti-saccades in the primate frontal eye field.. J Neurosci.

[27] Connolly JD, Goodale MA, Goltz HC, Munoz DP (2005). fMRI activation in the human frontal eye field is correlated with saccadic reaction time.. J Neurophysiol.

[28] O'Shea J, Muggleton NG, Cowey A, Walsh V (2004). Timing of target discrimination in human frontal eye fields.. J Cogn Neurosci.

[29] Taylor PC, Nobre AC, Rushworth MF (2007). FEF TMS affects visual cortical activity.. Cereb Cortex.

